# Fremanezumab Improved Migraine and Headache Attributed to Glioblastoma

**DOI:** 10.7759/cureus.30064

**Published:** 2022-10-08

**Authors:** Shin Kawamura, Masahito Katsuki, Kenta Kashiwagi, Akihito Koh

**Affiliations:** 1 Department of Neurosurgery, Itoigawa General Hospital, Itoigawa, JPN; 2 Department of Neurology, Itoigawa General Hospital, Itoigawa, JPN

**Keywords:** pain management, alternative medicine, migraine, palliative care, headache, glioblastoma, fremanezumab, anti-calcitonin gene-related peptide monoclonal antibodies

## Abstract

Fremanezumab, one of the anti-calcitonin gene-related peptide monoclonal antibodies, is widely used for migraine prophylaxis. However, its efficacy for headache attributed to glioblastoma has not been reported. We herein report a 66-year-old man who had right temporoparietal glioblastoma which recurred despite surgical and chemo-radiological treatment. He had migraine and headache attributed to glioblastoma, but fremanezumab improved both of them. Our case suggested that fremanezumab’s possible efficacy for not only migraine but also headache attributed to intracranial neoplasia.

## Introduction

Glioblastoma (GBM), a World Health Organization (WHO) grade IV astrocytoma, representing 15 to 20% of all primary intracranial neoplasms in adults, is highly aggressive, with an unusually dismal prognosis. Although the symptoms and prognoses depend on the pattern of GBM recurrence [1], the most common general symptom of GBM is headache [2], and headache related to GBM regrowth or recurrence reduces the quality of daily life.

Fremanezumab, one of the anti-calcitonin gene-related peptide (CGRP) monoclonal antibodies, is used for episodic migraines. Fremanezumab is a fully humanized IgG2Δa/kappa monoclonal antibody that potently and selectively binds to both isoforms of CGRP. Fremanezumab prevents episodic migraine in Japanese and Korean patients with no new safety concerns. Over 40% of patients reach at least a 50% reduction in the monthly average number of migraine days during the 12‐week period after initial administration of fremanezumab [3]. Approximately 1% of the elderly have migraine headaches [4], and preventive treatment of migraine is expected to become more common.

Still, its efficacy has not been reported for headaches attributed to intracranial neoplasia (International Classification of Headache Disorders 3rd edition (ICHD-3) code 7.4.1.). We report a case with GBM and migraine, whose headache attributed to intracranial neoplasia and migraine without aura (code 1.1) were both successfully treated by fremanezumab.

## Case presentation

**Figure 1:**
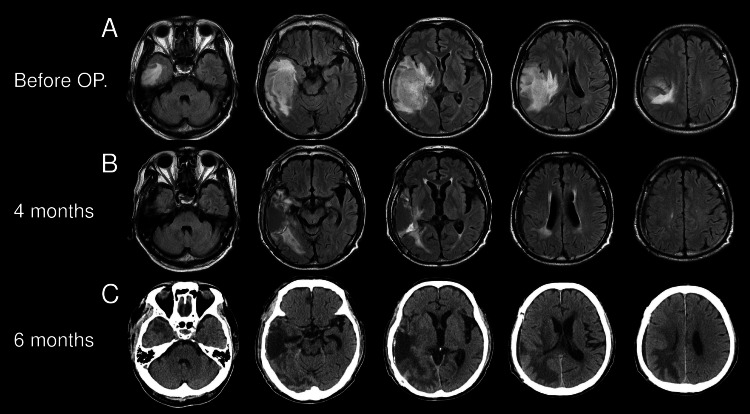
Radiological findings Fluid-attenuated inversion recovery revealed a 40 mm solid tumor with severe brain edema at the right temporoparietal lobe (A). After four months, the fourth maintenance therapy was performed. At this time, the residual tumor and edema were most improved (B). Six months later, the patient complained of headaches and nausea at the start of the sixth maintenance therapy session. The head computed tomography showed severe brain perifocal edema, suggesting regrowth or recurrence (C).

A 66-year-old right-handed man walked into our hospital with a headache of a numerical rating scale (NRS) of 4/10 and apraxia. The Glasgow Coma Scale was 14 (E4V4M6), and he had no paresis. The headache was not pulsating and was located in the right occipital region. Nausea was accompanied. He suffered from migraine from his teens to his 40s, but it was spontaneously relieved before. Four years ago, he had bilateral kidney removal due to ureteral cancer and was now on dialysis. Fluid-attenuated inversion recovery revealed a 40 mm solid tumor with severe brain edema at the right temporoparietal lobe (Figure 1A).

We diagnosed a primary brain tumor and performed surgical resection. We achieved partial removal considering the damage to the pyramidal tract. The pathological diagnosis was GBM, WHO grade IV, IDH wild-type. Based on the Stupp regimen, chemotherapy using temozolomide (75 mg/m^2^ per day) and radiotherapy (60 Gy/30Fr) were started. His headache was relieved, and his apraxia gradually improved. After that, maintenance therapy began with temozolomide (150 mg/m^2^ per day, five days) and bevacizumab (10 mg/kg) during each 28-day cycle.

After four months, the fourth maintenance therapy was performed. At this time, the residual tumor and edema were most improved (Figure 1B). There were no headaches, and he could walk with a cane and drive a car (Karnofsky performance status 90). However, six months after surgery, at the start of the sixth maintenance therapy, the patient complained of headache and nausea. The headache was not pulsating but fluctuating during the day and was located in the right occipital area with neck pain. The headache increases in the morning and improves with time but can be aggravated by stress or weather changes. It also worsens with physical activities, and the pain ranges from 3-10 on the NRS. It improves with oral acetaminophen. He said that the morning and daytime headaches seem different and that the headache that occurs during the day resembles the migraine headaches he had when he was younger. The head computed tomography showed severe brain edema, suggesting regrowth or recurrence of the GBM (Figure 1C).

Based on the history of headache and significant response to acetaminophen, we diagnosed migraine without aura (code 1.1) and headache attributed to intracranial neoplasia (code 7.4.1). Because the patient was on dialysis and was already taking anticonvulsants and antihypertensive medications, fremanezumab 675 mg was administered to prevent migraine attacks. From the next day, after getting up, daytime migraine attacks ceased to occur. The headache upon waking also improved, and nausea disappeared. It was about 12 h after the administration. After one month after the first fremanezumab administration, we performed the seventh time maintenance therapy but left mild paresis (4/5) rapidly appeared. Karnofsky performance status is now 50, but Hasegawa Dementia Scale-Revised was 29/30. Three months after fremanezumab administration, we performed the ninth time maintenance therapy. The left paresis (1/5) was seen, but the Glasgow Coma Scale was 15 (E4V5M6). He came to the hospital using a wheelchair. Karnofsky performance status was 40. During these three months, there were no headaches nor migraine attacks.

Our hospital’s research ethics committee approved this case report (ethical approval number 2021-4). We gained written informed consent for this study from the patient. All methods were carried out under relevant guidelines and regulations (Declaration of Helsinki). This article was previously posted to the Research Square preprint server on September 8, 2022.

## Discussion

Surgery, chemotherapy, and radiation therapy are all used in the multimodal treatment of headaches caused by intracranial neoplasia. We performed these therapies, although concurrent headaches due to GBM recurrence and migraines were experienced. Since nonsteroidal anti-inflammatory medicines are ineffective for treating headache caused by elevated intracranial pressure, hyperosmotic fluids like concentrated glycerin, fructose, or D-mannitol, steroids, and cerebral edema are typically utilized to treat the condition [5]. The management of headaches is important because headaches exacerbate the patient’s quality of life. In our case, chemotherapy with temozolomide and bevacizumab could not improve his headache. However, fremanezumab was administered for migraine, which resulted in improvement of both headaches attributed to GBM and migraine.

The mechanism of headache attributed to intracranial neoplasia has not been clarified. The dural branches of the trigeminal nerve, facial nerve, glossopharyngeal nerves, vagus nerve, and branches of the superior cervical nerve are distributed in the intracranial and extracranial dura mater, scalp, fascia of the head, muscles, periosteum, and arteriovenous vessels. Therefore, the pain due to stimulation of dura mater or vessels may appear in the temporal and occipital regions. The vagus nerve, hypoglossal nerve, and the first and second cervical nerves innervate the dural branches and dural arteries of the posterior cranial fossa, and their associated pain may appear in the posterior neck area. The falx and supraorbital walls are innervated by the first branch of the trigeminal nerve, and the middle cranial fossa is innervated by the second branch of the trigeminal nerve. These painful nerves may be tractioned or compressed by increased intracranial pressure due to glioma. The tumor may also cause headaches due to the deviation of cerebral blood vessels and traction or direct compression of painful nerves, individually or in conjunction with each other [6].

One of the possible mechanisms is attenuating trigeminal nerve sensitivity. Considering that the mechanism of headache attributed to intracranial neoplasia is based on this physical stress for painful nerves, which most branched from the trigeminal nerve, a similar context that CGRP antibody improves trigeminal neuralgia may help in understating the pathophysiology of headache attributed to intracranial neoplasia. The efficacy of the CGRP antibodies for trigeminal neuralgia has been reported [7]. Those with trigeminal neuralgia have higher serous CGRP levels, and CGRP sensitizes afferent neurons of the trigeminal nerve, even in the absence of prior inflammation. This supports the idea that CGRP released from intraganglionic regions may mediate pain transmission, presumably by releasing various cytokines [7]. In rats, the intracisternal injection of CGRP made them more sensitive to sensory stimuli and enhanced their withdrawal from pain. A CGRP antagonist prevented the increase of this reaction [8]. Therefore, CGRP may have a role in the trigeminal nerve, and CGRP antagonist may blunt the pain caused by sensory stimulation of the head, leading to the attempt to treat trigeminal neuralgia with an anti-CGRP monoclonal antibody. Given that both trigeminal neuralgia and headache attributed to intracranial neoplasia are ultimately perceived as pain via the trigeminal ganglion, suppression of CGRP in the trigeminal ganglion may have led to pain relief.

The other possible mechanism is attenuating pain signaling in other sites in the brain than the trigeminal nerve. CGRP and CGRP receptors have been found in other peripheral and CNS sites involved in pain signaling, including the striatum, amygdala, hypothalamus, thalamus, and brainstem. In particular, thalamic modulation has identified an inhibitory effect of CGRP antagonism on nociceptive signaling. CGRP may also play a role in mediating pain signals from some Aẟ and C fibers [9]. This pain sensitivity modulation is another possible mechanism. Also, the headache may be aggravated or modified by promoting systemic inflammation due to GBM [10], which is related to GBM prognosis.

## Conclusions

We herein report a 66-year-old man who had right temporoparietal glioblastoma which recurred despite surgical and chemo-radiological treatment. He had migraine and headache attributed to glioblastoma, but fremanezumab improved both of them. Given that headaches attributed to intracranial neoplasia are ultimately perceived as pain via the trigeminal ganglion, suppression of CGRP in the trigeminal ganglion may have led to pain relief. Our case suggested fremanezumab’s possible efficacy for not only migraine but also headache attributed to intracranial neoplasia. Fremanezumab may help improve the quality of life in end-of-life care among brain tumor patients, where headaches are often neglected.
